# Multiple Myeloma and B Cell Lymphoma. Investigation of IL-6, IL-6 Receptor Antagonist (IL-6RA), and GP130 Antagonist (GP130A) Using Various Parameters in an *In Vitro* Model

**DOI:** 10.1100/tsw.2006.178

**Published:** 2006-08-03

**Authors:** Eva Kovacs

**Affiliations:** Society of Cancer Research, Arlesheim, Switzerland

**Keywords:** multiple myeloma, B cell lymphoma, IL-6, IL-6R, gp130, proliferation, cell cycle

## Abstract

Interleukin-6 (IL-6) affects the survival and proliferation of myeloma cells via autocrine and/or paracrine mechanisms. In this study, we investigated the effects of IL-6, IL-6 receptor antagonist (IL-6RA), and gp130 antagonist (gp130A) on the membrane expressions of IL-6R and gp130, on the viability, on the proliferation, on the DNA synthesis, and on the cell cycle phases in several multiple myeloma (MM) cell lines and B cell lymphoma cell lines. Our results showed that (1) all five MM cell lines (OPM-2, RPMI-8226, U-266, KMS-12-BM, MOLP-8) expressed surface IL-6R and gp130, the B cell lymphomas (WSU-1, DOHH-2, U-698) expressed only gp130; (2) exogenous IL-6 markedly up-regulated the expression of membrane IL-6R (up to 186%) and down-regulated the gp130 receptor (down to 4%) in MM cell lines, the membrane expression of gp130 in B cell lymphomas was not altered; (3) IL-6 markedly increased the spontaneous proliferation (up to 151%) in all MM cell lines, that of B cell lymphomas was not affected; (4) IL-6 increased the DNA synthesis in the S cell cycle phase of MM cells and arrested the stage G2/M, IL-6 was ineffective in any cell cycle phase of B cell lymphoma; (5) IL-6RA inhibited the membrane IL-6R partially, the proliferation was decreased only slightly; and (6) although gp130A inhibited the membrane gp130 completely, the proliferation was decreased 81—78% in MM and B cell lymphoma cell lines. This means that gp130 is not absolutely necessary for the cellular signalling cascade via JAK/STAT and RAS/MAPK pathways involved in proliferation and viability. Our results give an indication in the therapy of MM: IL-6 antibody (IL-6A) alone or in combination with IL-6RA. The latter could be more effective. This kind of therapy is not recommended for B cell lymphoma, as these cells have no IL-6R.

## INTRODUCTION

Lymphomas are mainly malignant diseases of B cells. Multiple myeloma (MM) is a haematological disorder of clonal malignant plasma cells that accounts for 1–2% of all human cancers. Many cytokines are involved in the growth and survival of these tumour cells[1,2]. IL-6 has been identified as one of the most important cytokines for the myeloma cells. It enhances survival of myeloma cells because it inhibits apoptosis by induction of the anti-Fas[3]. MM cells proliferate via autocrine or paracrine mechanisms[4,5]. IL-6 with a molecular weight of 21–30 kDa mediates its function through a receptor composed of two membrane subunits: an $\alpha$ chain (the IL-6 binding protein IL-6R [80 kDa]) and a $\beta$ chain (the signal transducer gp130 [130 kDa]). This complex could lead to an activation of JAKs/STAT and RAS/MAPKs pathways. Janus kinases (JAKs) and the signal transducer-activator of transcription (STAT) regulate survival. RAS/mitogen-activated protein kinase (MAPK) is involved in the proliferation. The gp130 molecule is the common signal transducer for receptors of IL-6, IL-11, leukaemia inhibitory factor (LIF), Oncostatin M (OM), and ciliary neurotrophic factor (CNTF)[6]. All these cytokines could be important factors in the survival and proliferation of the MM cells. Gp130 is expressed in T cells, activated B cells, monocytes, plasma cells, and endothelia, suggesting that this glycoprotein may also be used as a signal transducer for some other cytokines.

In a previous study, we found that exogenous IL-6, although it up-regulated the membrane receptor of IL-6, markedly inhibited the surface expression of gp130 in one human MM cell line, OPM-2. Nevertheless, the IL-6/IL-6R complex without gp130 led to an increased proliferation of the tumour cells and survival was not impaired[4]. This finding was unexpected. Therefore, it was of interest to investigate the effects of exogenous IL-6 in other tumour cell lines.

Additionally, we also tested IL-6 receptor antagonist (IL-6RA) and gp130 antagonist (gp130A). IL-6RA specifically recognizes the human IL-6R and gp130A recognizes the IL-6-receptor-associated signal transducer gp130. As parameters, we measured the viability, the membrane expression of IL-6R and gp130, the proliferation of tumour cell lines, the DNA synthesis, and the different cell cycle phases of these cells. We used MM cell lines (RPMI-8266, U-266, OPM-2, KMS-12-BM, MOLP-8) and B cell lymphoma cell lines (DOHH-2, WSU-1, U-698).

## MATERIALS AND METHODS

### Test Substances

Recombinant human interleukin-6 (rh IL-6) was obtained from R&D Systems (No 206, U.K.) and reconstituted in phosphate-buffered saline (PBS) with 0.18% bovine serum albumin. The IL-6 was calibrated with the NIBSC/WHO international standard (1 µg IL-6 is equivalent to $1.1 \times 10^5$ IU. Human IL-6RA was obtained from MedSystems (No BMS 135, Diagnostics GmbH, Austria) and reconstituted in PBS. IL-6RA can be used for functional studies and for FACs analysis to determine the expression of membrane receptor. Human gp130A was obtained from PharMingen (No 555755; BD Biosciences, Germany) and reconstituted in PBS. Gp130A is useful for *in vitro* functional studies and for flow cytometric analysis.

### Cells and Culture Condition

The human MM cell lines (OPM-2, RPMI-8226, U-266, KMS-12-BM, MOLP-8) and the B cell lymphoma cell lines (WSU-1, DOHH-2, U-698) were obtained from DSMZ (Braunschweig, Germany). The cells were cultivated in RPMI 1640 supplemented with 15% foetal calf serum, 2 m*M* L-glutamine, and 1% gentamicin in a humidified atmosphere with 5% $CO_2$ at 37°C. The doubling times of the tumour cells were 48 h for OPM-2, 60 h for U-266, 50 h for RPMI-8226, 40 h for MOLP-8, 60 h for KMS-12-BM, 45 h for WSU-1 and DOHH-2, 48 h for U-698. To measure parameters, the cell cultures were used within 4 weeks after thawing.

## Treatment of the Cells with IL-6, IL-6RA, and gp130A to Measure Viability, Membrane Expression of Receptors and Cytokine Production

The cells were cultivated at a density of 0.6–0.7 × $10^6$ cells/ml in six-well plates. The cells were incubated for different times with (1) IL-6 (5 ng/$10^6$ cells/ml), (2) IL-6RA (0.2 μg/$10^6$ cells/ml), and (3) gp130A (10 μg/$10^6$ cells/ml).

## Viability

The viabilities of the cultivated tumour cells 24 and 72 h after incubation were determined by 7-amino-actinomycin D (7-AAD), to exclude the nonviable cells in flow cytometric assays. The values are given in percent.

## Measurement of Membrane Expressions of IL-6R and gp130

The cells were incubated for 24 h. For immunofluorescence staining, 3 × $10^5$ cells/100 μl were incubated with 20-μl phycoerithrin (PE)-conjugated monoclonal antibodies (CD 126: Immunotech, France and CD 130 BDPharmingen ) for 30 min at 4°C. Then the cells were washed, sedimented, and analysed in the FACSCalibur flow cytometer. For the expression of the membrane IL-6R (CD 126) and gp130 (CD 130), the signal intensity (geometric mean of the fluorescence intensity × counts) was used as parameter. The signal intensity for each marker and each sample was compared with those of untreated samples, which were taken as 100%.

## Cytokine Production

The spontaneous IL-6 productions in the supernatant of the cultivated cells were determined by chemiluminescent immunometric assay. The lowest detectable levels were 2 pg/ml.

## Measurements of the Proliferation

The cells were treated with IL-6 (0.5 ng/$10^5$ cells/100 μl) for 72 h or with IL-6RA (0.02 μg/$10^5$ cells/100 μl) or gp130A (1 μg/$10^5$ cells/100 μl) for 24 h after 48 h of cultivation. The proliferation rate of the myeloma and the lymphoma cell lines was measured 4 h after incubation using cell proliferation reagent WST-1 (Roche, Mannheim, Germany, Cat. No. 1644 807).

## Measurements of the Cell Cycle Phases

The cells of RPMI-8226 and WSU-1 were treated for 24, 48, or 72 h with (1) IL-6 (5 ng/$10^6$ cells/ml), (2) IL-6RA (0.2 μg/$10^6$ cells/ml). IL-6RA was added 24 h before measurement at each time point. The cell cycle phases G0/G1, S, G2/M were assessed using the cycle test plus DNA reagent kit on a flow cytometer. 5 × $10^5$ cells were incubated at room temperature with trypsin buffer and additionally with trypsin inhibitor + RNase buffer. After addition of propidium iodide, the samples were analysed on a flow cytometer.

## Measurement of DNA Synthesis

The DNA synthesis of the MM cell line RPMI-8226 was measured by the incorporations of BrdU (5-bromo-2-deoxyuridine) in the proliferating cells. Six wells were used for untreated or treated cells.

Briefly, the cells were treated with IL-6 (0.5 ng/$10^5$ cells/100 µl) or with IL-6RA (0.02 µg/$10^5$ cells/100 µl) for 48 or 72 h. IL-6RA was added 24 h before measurement. Thereafter, the cells were incubated with 1 µ*M* BrdU for 6 h. The developed colour and thereby the absorbance values directly correlate to the amount of DNA synthesis.

### Statistical Analysis

Three or five repeated measurements were carried out. For the evaluation of the parameters, the Mann Whitney U-test was used. The limit of significance was taken as $p < 0.05$.

## RESULTS

### Viability of Tumour Cells

The viabilities of control cells without test substances lay in the range of 71–95% during 72 h of incubation. The test substances (IL-6, IL-6RA, gp130A) did not impair the viability of the cells (results not shown).

### Membrane Expression of Surface Receptors

Table 1 presents the signal intensities as the parameter for the expressions of surface IL-6R and gp130 in MM cell lines (OPM-2, RPMI-8226, U-266) and in B cell lymphoma lines (WSU-1, DOHH-2) treated for 24 h with IL-6, IL-6RA, or gp130A. The values of four independent experiments are presented as mean ± SEM, and are also expressed in percentage of the untreated samples. For RPMI-8226, Fig. 1 presents the kinetics of the membrane expression of IL-6R and gp130. The parameters were investigated in two independent experiments at different times (1, 4, 12, 24 h) during incubation with IL-6 (5 ng/$10^6$ cells/ml), IL-6RA (0.2 µg/$10^6$ cells/ml), gp130A (10 µg/$10^6$ cells/ml), IL-6RA + IL-6, and gp130A + IL-6. For the latter, IL-6RA or gp130A was added 2 h before IL-6 at each time point.

### *Expression of IL-6R*

#### MM Cell Lines

The expressions of IL-6R in the three untreated cells are in the similar range (Table 1). Exogenous IL-6 significantly up-regulated the membrane expression of its receptors in all MM cell lines (range 133–186%), ($p < 0.05$ and $p < 0.01$). IL-6RA inhibited the membrane receptor expression down to 51–83% ($p < 0.05$ and $p < 0.01$). Higher doses of IL-6RA (0.4, 0.8, 1, and 2 µg/$10^6$ cells) did not alter the receptor expression (results are not presented). Gp130A did not affect the surface IL-6R.

The time course using MM cell lines RPMI-8226 (Fig. 1) shows that the expression of the membrane receptor of IL-6 is markedly up-regulated after 12 and 24 h of incubation with exogenous IL-6. IL-6RA decreased the receptor expression down to 25% during 24-h incubation. With IL-6RA + IL-6, the kinetics curve of membrane expression was similar, but values were lower than with IL-6; they did not reach the level of the untreated samples. With gp130A, the expression of surface IL-6R was not altered. With gp130A + IL-6, the kinetics curve and the levels of the values were the same as those of IL-6.

**TABLE 1**
**The Effects of IL-6, IL-6RA, and gp130A on the Expression of the Surface IL-6R and Membrane Protein gp130 in Human MM and B Cell Lymphoma Cell Lines**

| Treatment Cell Lines | Untreated Samples | | IL-6 (5 ng/$10^6$ Cells) | | IL-6RA (0.2 μg/$10^6$ Cells) | | gp130A (10 μg/$10^6$ Cells) | |
|---|---|---|---|---|---|---|---|---|
| | IL-6 R | gp130 | IL-6 R | gp130 | IL-6 R | gp130 | IL-6 R | gp130 |
| **MM** | | | | | | | | |
| OPM-2 | 568,305 ± 24,717 | 101,212 ± 3,187 | 927,146 ± 27,037[a] (163%) | 4,267 ± 913[b] (4%) | 402,553 ± 24,802 (71%) | 100,003 ± 7,464 (99%) | 562,125 ± 20,100 (99%) | 304 ± 9 (0.3%) |
| RPMI-8226 | 420,688 ± 85,446 | 588,311 ± 127,065 | 781,793 ± 177,667[b] (186%) | 155,741 ± 31,329[b] (26%) | 214,708 ± 41,872 (51%) | 585,523 ± 129,102 (99%) | 416,203 ± 80,012 (99%) | 2,941 ± 78 (0.5%) |
| U-266 | 697,726 ± 20,383 | 114,308 ± 5,679 | 927,074 ± 46,350[a] (133%) | 20,975 ± 1,541[b] (17%) | 579,195 ± 9,491 (83%) | 107,700 ± 4,828 (95%) | 682,120 ± 170,502 (98%) | 114 ± 10 (0.1%) |
| **B Cell** | | | | | | | | |
| WSU-1 | ND | 11,251 ± 515 | ND | 12,138 ± 450 | ND | 11,920 ± 482 | ND | ND |
| DOHH-2 | ND | 119,296 ± 8,150 | ND | 123,808 ± 12,100 | ND | 130,733 ± 9,500 | ND | ND |

The cells were incubated for 24 h. The results of four independent experiments are presented as mean ± SEM. For the expression of the membrane IL-6R and gp130, the signal intensity was taken as parameter. The percentage of untreated samples was taken as 100%.

[a]$p$ < 0.05; [b]$p$ < 0.01 compared with untreated samples; ND, not detectable.

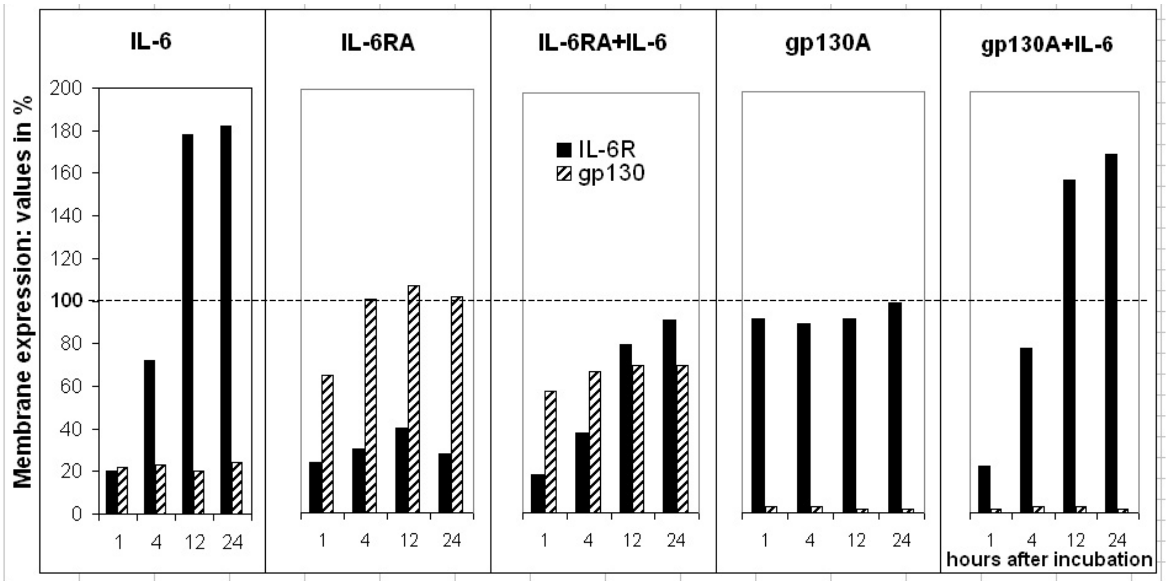

**FIGURE 1.** The effects of IL-6, IL-6RA, IL-RA + IL-6, gp130A, and gp130A + IL-6 on the membrane expression of IL-6R and gp130 in RPMI-8226 human MM cell line. In two independent experiments, the cells were cultivated for different times with 5 ng/$10^6$ cells/ml of recombinant human IL-6 or with 0.2 μg/$10^6$ cells/ml of human IL-6RA or with 10 μg/$10^6$ cells/ml of gp130A or with a combination of these. The mean values of the signal intensity are expressed as percentage of the untreated samples (100%).

### B Cell Lymphoma Cell Lines

The B cell lymphomas WSU-1 and DOHH-2 did not express surface IL-6R (Table 1), therefore IL-6RA and gp130A had no effect.

## *Expression of gp130 Receptor*

All three MM cell lines and the two B cell lymphoma cell lines expressed surface gp130. The membrane expressions were markedly different in the untreated MM cell lines and in the B cell lymphomas (Table 1). Exogenous IL-6 down-regulated the expression of surface protein gp130 4–26% for the MM cell lines ($p < 0.01$); the membrane expression for WSU-1 and DOHH-2 was not impaired. IL-6RA did not affect membrane expression in either MM cell lines or in B cell lymphomas. Gp130A inhibited the expression of surface gp130 completely (ND–0.5%) in both tumour cell lines.

As the time course in RPMI-8226 shows, the values were lowered down to 20% by IL-6 at 1, 4, 12, and 24 h. The membrane expression of gp130 was not inhibited by IL-6RA. When IL-6RA + IL-6 was added to the cell cultures, the membrane expression was inhibited about by 30% at each time point. With gp130A and gp130 + IL-6, the expression of surface protein gp130 was inhibited completely (0.3%) during 1, 4, 12, and 24 h.

## Production of IL-6 by the Tumour Cells

None of the MM tumour cell lines or B cell lymphoma lines produced IL-6 spontaneously (results not shown).

## Proliferation of Tumour Cells

Fig. 2 shows the proliferative effects of exogenous IL-6, IL-6 RA, and gp130A in three MM cell lines and in two B cell lymphoma cell lines after 72-h incubation. The values are expressed as percentage of the untreated samples and are the average of three or four independent experiments. IL-6 led to increases of proliferation in RPMI-8226 (up to a mean of 160%), in OPM-2 (up to a mean of 132%), and in U-266 (up to a mean of 124%) ($p < 0.05$). When the surface IL-6R was inhibited by its antagonist (IL-6RA), the spontaneous proliferation of the MM cells slightly decreased below that of the untreated samples (NS). Exogenous IL-6 or IL-6RA did not affect the spontaneous proliferation of B cell lymphomas WSU-1 and DOHH-2. Gp130A inhibited the spontaneous proliferation in all three MM cell lines and in two B cell lymphoma cell lines (down to 81–78%). We found a significance ($p < 0.05$) in WSU-1, DOHH-2, and in OPM-2 tumour cell lines.

## Cell Cycle Phases of Tumour Cell Lines RPMI 8226 and WSU-1

Fig. 3 shows the cell cycle phases G0/G1, S, G2/M of myeloma cells RPMI-8226 and B cell lymphoma WSU-1. The cells were incubated with IL-6 (5 ng/$10^6$ cells/ml) or IL-6RA (0.2 μg/$10^6$ cells/ml) for 24, 48, and 72 h. IL-6RA was added 24 h before measurement. The values are the mean + SD of four independent experiments, expressed in percentage of total cell number. The number of cells with the least DNA content is much greater in the G0/G1 phase than the number of cell with the highest DNA content in the phases S and G2/M (Fig. 4).

**RPMI-8226**: The numbers of the untreated cells increased in phase G0/G1 but decreased in cycle phases S and G2/M during 72-h cultivation. With IL-6, the number of cells in phase G0/G1 was lower than in untreated cells. In contrast, IL-6 clearly increased the number of cells in cycle phases S and G2/M. With IL-6RA, the percentage of the cells in the different cycle phases was the same as that of untreated cells at each investigated time.

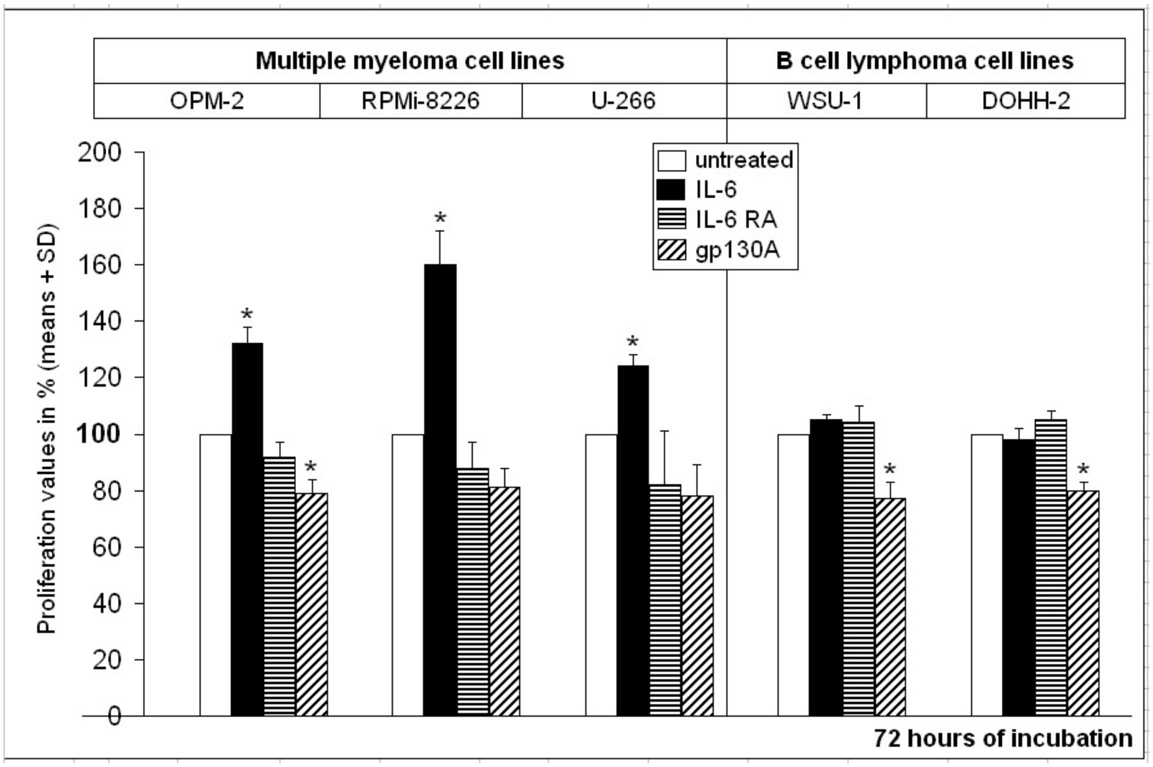

**FIGURE 2.** The proliferative response of human tumour cell lines to IL-6, IL-6RA, or gp130A. The cells were incubated with IL-6 (0.5 ng/$10^5$ cells/100 µl) for 72 h or with IL-6RA (0.02 µg/$10^5$ cells/100 µl), or gp130A (1 µg/$10^5$ cells/100 µl) for 24 h after 48 h of cultivation. The proliferation was measured by WST-1 reagent. The results of three or four independent experiments (mean + SD) are expressed as percentage of the untreated samples. *$p < 0.05$ (Mann Whitney U-test) vs. untreated.

**WSU-1**: Without treatment, the number of cells increased in the G0/G1 phase and decreased in the phases S and G2/M during 72-h cultivation. We did not find an alteration with IL-6 treatment in any cycle phase.

## DNA synthesis of tumour cell lines RPMI 8226 and WSU-1

Table 2 presents the values of absorbance and percentage for RPMI-8226 after 48 or 72 h of incubation with IL-6 or IL-6RA in comparison with untreated samples. IL-6 led to increased values (up to 137%) in MM cell line RPMI-8226. With IL-6RA, the values lay in the range of untreated samples.

## DISCUSSION

Multiple myeloma (MM) is characterised as a malignant plasma cell proliferation. Many cytokines are involved in the growth of the tumour cells and the progression of the disease. IL-6 is a major growth factor for malignant plasma cells. It affects the cells mainly by an autocrine mechanism with an additional paracrine signalling. Using several human MM cell lines and human B cell lymphoma cell lines, we have investigated the proliferation, the viability, the expressions of the surface receptor IL-6 (IL-6R) and the membrane protein gp130, the cell cycle phases, and the DNA synthesis.

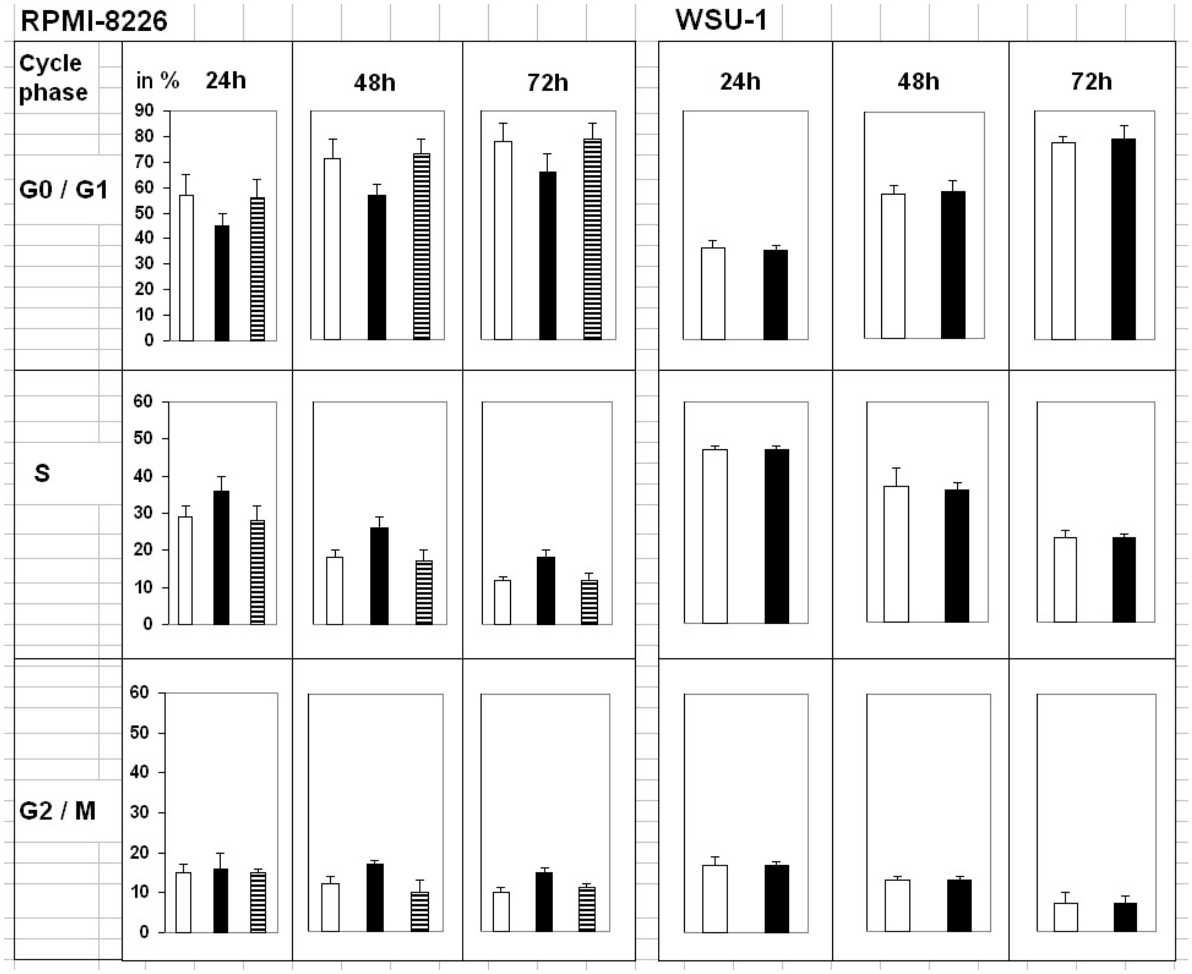

**FIGURE 3.** Cell cycle analysis of MM cell line RPMI-8226 and B cell lymphoma cell line WSU-1. The cells were incubated with IL-6 (5 ng/$10^6$ cells/ml) or with IL-6RA (0.2 µg/$10^6$ cells/ml) for 24, 48, or 72 h. IL-6RA was added 24 h before. The cell cycle phases G0/G1, S, G2/M were assessed using the cycle test plus DNA reagent kit. The values are the mean + SD of four independent experiments expressed in percentage of total cell number. Untreated (Open), IL-6 (Closed), IL-6RA (Cross-hatch).

All investigated cell lines proliferated spontaneously with a doubling time between 40–60 h; IL-6 was not detectable in the supernatant of any of these cultivated cell lines. This means that they have an IL-6 dependent or an IL-6 independent regulation mechanism. IL-6 acts via its specific membrane receptor IL-6R. All the three MM cell lines (OPM-2, RPMI-8226, U-266) expressed IL-6R receptor on the membrane, whereas the two B cell lymphomas (WSU-1, DOHH-2)) did not (Table 1). Additionally, we tested two MM (MOLP-8 and KMS-12-BM) and one B cell lymphoma (U-698) cell lines. We confirmed that only MM cell lines expressed membrane IL-6 receptors (results are not shown). This means that MM cell lines have an IL-6 dependent and the B cell lymphoma cell lines an IL-6 independent regulation mechanism. Tupitsyn et al.[7] described that the majority of B cell tumours expressed either IL-6R or gp130. Tomeczkowski et al.[8] and Klein et al.[9] found IL-6 receptors only in B cell lymphoma cell lines infected by EBV. The membrane protein gp130 was expressed in all investigated MM cell lines and all B cell lymphoma cell lines. The surface expressions of IL-6R lie in a similar range in all cell lines in contrast to gp130 (Table 1). The large variation in the membrane expression of this protein between the tumour cell lines could be due to its multifunctionality.

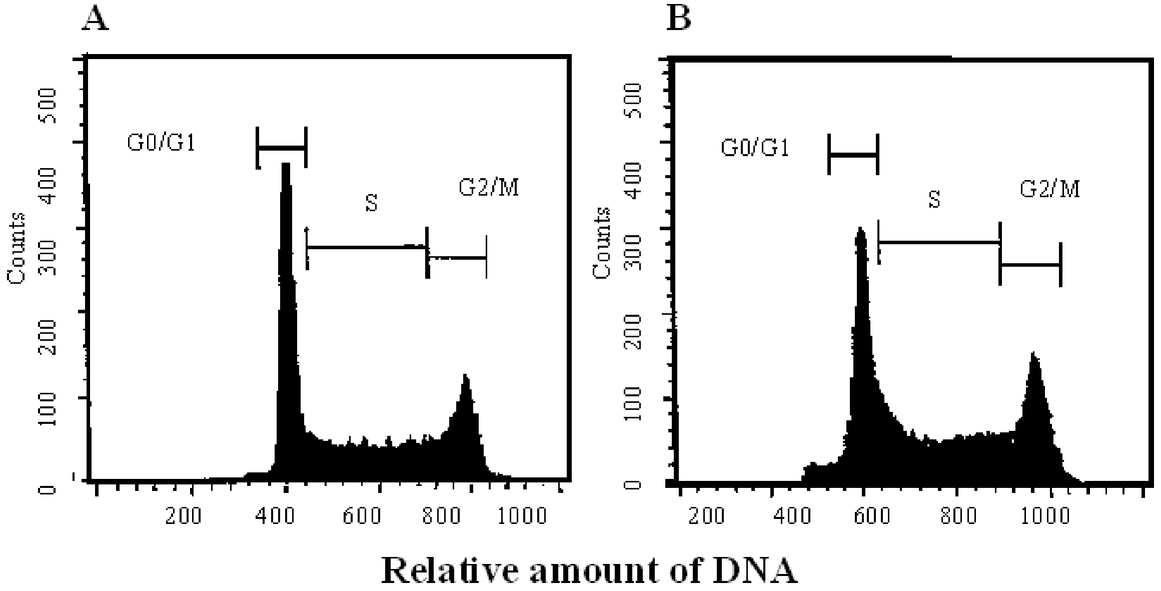

**FIGURE 4**. Histograms of the cultivated cells without (A) or with IL-6 (B) treatment in human myeloma cell line RPMI-8226. The X-axis represents the relative amount of DNA, the y-axis the number of cells. The number of cells with the least DNA content is much greater in the G0/G1 phase than the number of cells with the highest DNA content in the phase G2/M.

**TABLE 2**
**Effects of IL-6 and IL-6RA on the DNA Synthesis of MM RPMI-8226 Cells**

| Treatment | Incubation Time | |
|---|---|---|
| | 48 h Absorbance (in %) | 72 h Absorbance (in %) |
| Untreated | 0.65 (100) | 0.83 (100) |
| IL-6 (0.5 ng/$10^5$ cells) | 0.89 (137) | 1.1 (132) |
| IL-6 RA (0.02 µg/$10^5$ cells) | 0.6 (92) | 0.88 (106) |

The means of three experiments are presented.

In this study, IL-6 led to significant up-regulation of its surface receptor in all MM cell lines, confirming the findings in an earlier study[4]. The data in the literature are contradictory: Lasfar et al.[10] found a reduced membrane expression of IL-6R for RPMI-8226, Thabard et al.[11] an up-regulation for OPM-2.

Many authors report that IL-6 acts on the target cells via the surface IL-6R and the gp130 membrane protein. The complex IL-6/IL-6R/gp130 could activate the JAK/STAT and RAS/MAPK pathways[12]. In all MM cell lines investigated in our study, the proliferation was markedly increased by exogenous IL-6, although at the same time the gp130 membrane protein was markedly down-regulated (Fig. 1, Table 1). We assume that in this case there are still enough uninhibited membrane protein receptors for the cell to proliferate. In fact, when the IL-6 receptors were inhibited by IL-6RA (down 83–51 %), the proliferation was decreased only slightly (NS). Using the gp130A, the spontaneous proliferation decreased 81–78% in all three MM cell lines and in the two B cell lymphoma cell lines (Fig. 2), although gp130 membrane protein was inhibited completely. This means that gp130 is not absolutely necessary for the cellular signalling cascade via JAK/STAT and RAS/MAPK pathways involved in proliferation and viability. Our findings are therefore in contrast to the previously accepted role of IL-6/IL-6R/gp130. We postulate that there are two different proliferative pathways; either the duo IL-6/IL-6R or the trio IL-6/IL-6R/gp130. Is the duo an emergency pathway in the absence of gp130? Further studies could answer this question.

Cell division consists of two processes: mitosis (M) and interphase, which divides into phases G1, G2, S. Cells that are not dividing are in the stable resting phase, called the G0 phase. In our study, the number of untreated MM cells and B lymphoma cells increased in the G0/G1 phase during the 72 h, in contrast to the cell numbers in the S and G2/M cycle phases (Fig. 3). With our method, we cannot distinguish between cell cycle phases G0 and G1. It is therefore possible that there are more cells in the resting phase (G0) at 72 h. In all tumour cells, the spontaneous proliferation rate was lower at 72 h than at 24 h (results are not shown). With IL-6, the number of MM cells was increased in the S phase and in the G2/M phase compared with untreated cells. This means that either the DNA synthesis of the cells is increased or the cells are arrested in these cycle phases. The results of the DNA synthesis (Table 2) indicate that IL-6 led to an increased DNA synthesis in the S phase and possible arrest in the phase G2/M. Mori et al.[13] and Florenes et al.[14] found an arrest in the G1 phase of different tumours cell lines treated with IL-6; Cote et al.[15] arrested G1 cells in murine plasmacytoma with IL-6.

To inhibit the biological activity of the IL-6, monoclonal antibodies against IL-6 or IL-6R are used in the treatment of lymphoproliferative disorders. Our results show that MM cell lines are IL-6 dependent (autocrine mechanism with paracrine signalisation) and express surface IL-6R, whereas B lymphoma cells are IL-6 independent without expression of membrane IL-6R. According to the literature, only B lymphoma cells infected with EBV express membrane IL-6R. We have now shown that IL-6, together with its stimulated receptor, has a key role in the proliferation and in the cell cycle of MM tumour cells. Gp130 has a subsidiary position in the proliferation. Our results give an indication for the treatment of MM and in B lymphoma cells that are infected by EBV: either IL-6A alone or in combination with IL-6RA. The latter could be more effective. Gp130A is not recommended because it does not affect the surface IL-6R.

It is known that some other malignant tumours produce IL-6. Do these tumours express membrane IL-6R and are they IL-6 dependent? These questions will be addressed in further studies.

## ACKNOWLEDGEMENTS

The author thanks Dr. H. Langemann for the correction of this manuscript, Mrs. S. Link and Mrs. U. Toffol-Schmidt for the excellent technical assistance, and A. Schötzau for the statistical analysis.
